# SipB-SipC Complex Is Essential for Translocon Formation

**DOI:** 10.1371/journal.pone.0060499

**Published:** 2013-03-27

**Authors:** Sebenzile K. Myeni, Lu Wang, Daoguo Zhou

**Affiliations:** Department of Biological Sciences, Purdue University, West Lafayette, Indiana, United States of America; University of Louisville, United States of America

## Abstract

The delivery of effector proteins by *Salmonella* across the host cell membrane requires a subset of effectors secreted by the type III secretion system (TTSS) known as translocators. SipC and SipB are translocator proteins that are inserted into host membranes and presumably form a channel that translocates type III effectors into the host cell. The molecular events of how these translocators insert into the host cell membrane remain unknown. We have previously shown that the SipC C-terminal amino acid region (321–409) is required for the translocation of effectors into host cells. In this study, we demonstrate that the ability to form SipC-SipB complex is essential for their insertion into the host membrane. The SipB-interacting domain of SipC is near its C-terminal amino acid region (340–409). In the absence of SipB, SipC was not detected in the membrane fraction. Furthermore, SipC mutants that no longer interact with SipB are defective in inserting into the host cell membrane. We propose a mechanism whereby SipC binds SipB through its C-terminal region to facilitate membrane-insertion and subsequent translocon formation in the host cell membrane.

## Introduction

Many gram negative bacterial pathogens utilize type three secretion systems (TTSS) to translocate virulence factors into host cells to exploit host-cell functions for their own benefit. *Salmonella enterica* serovar Typhimurium (*S. typhimurium*), use the TTSS to cause gastroenteritis and typhoid fever in both humans and animals by delivering type III effectors into host cells [1], [2], [3], [4], [5]. The transport of bacterial effector proteins across the host cell membrane is facilitated by a subset of *Salmonella* effector proteins that are known as translocators: SipB, SipC, and SipD. These translocators presumably form a channel in the host cell membrane that enables the passage of bacterial effectors across the host cell membrane [6], [7], [8], [9]. The functional equivalents of *Salmonella* SipB, SipC and SipD in other pathogenic bacteria include; IpaB, IpaC and IpaD in *Shigella* spp.; YopB, YopD and LcrV in *Yersinia* spp.; PopB, PopD and PcrV for *Pseudomonas* spp.; EspA, EspD, and EspB in Enteropathogenic *Escherichia coli* (*EPEC*) [10], [11], [12].

SipB and SipC associate with artificial membranes and localize to the host cell membrane through their putative hydrophobic regions [13], [14]. All three translocators, SipB, SipC and SipD, are required to form functional pores during *Salmonella* infection of erythrocytes [15]. SipC, SipB, and SipD were further shown to be required for the intimate association of *Salmonella* with mammalian cells [16]. The intimate attachment of *Salmonella* with host cells is believed to trigger the TTSS while the insertion of SipC and SipB into membranes and pore formation are conceived to be important for protein translocation into host cells. The oligomerization of SipC is believed to be essential for the translocon pore formation [17]. In addition, it has been shown that SipC forms an extracellular complex with SipB during secretion [14], [18]. It was speculated that the SipB-SipC complex is important for the channel formation and the translocation of effectors into the host cell. However, how SipB and SipC insert into the host cell membrane and how the channels are formed remains unknown. In this study, we present evidence that the complex formation between SipC and SipB is essential for the insertion of both proteins into the host cell membrane during *Salmonella* invasion. We propose a mechanism whereby SipC binds SipB through its C-terminal region to facilitate subsequent translocon formation.

## Results

### Pore forming activities of SipC C-terminal mutant strains

**Figure 1:**
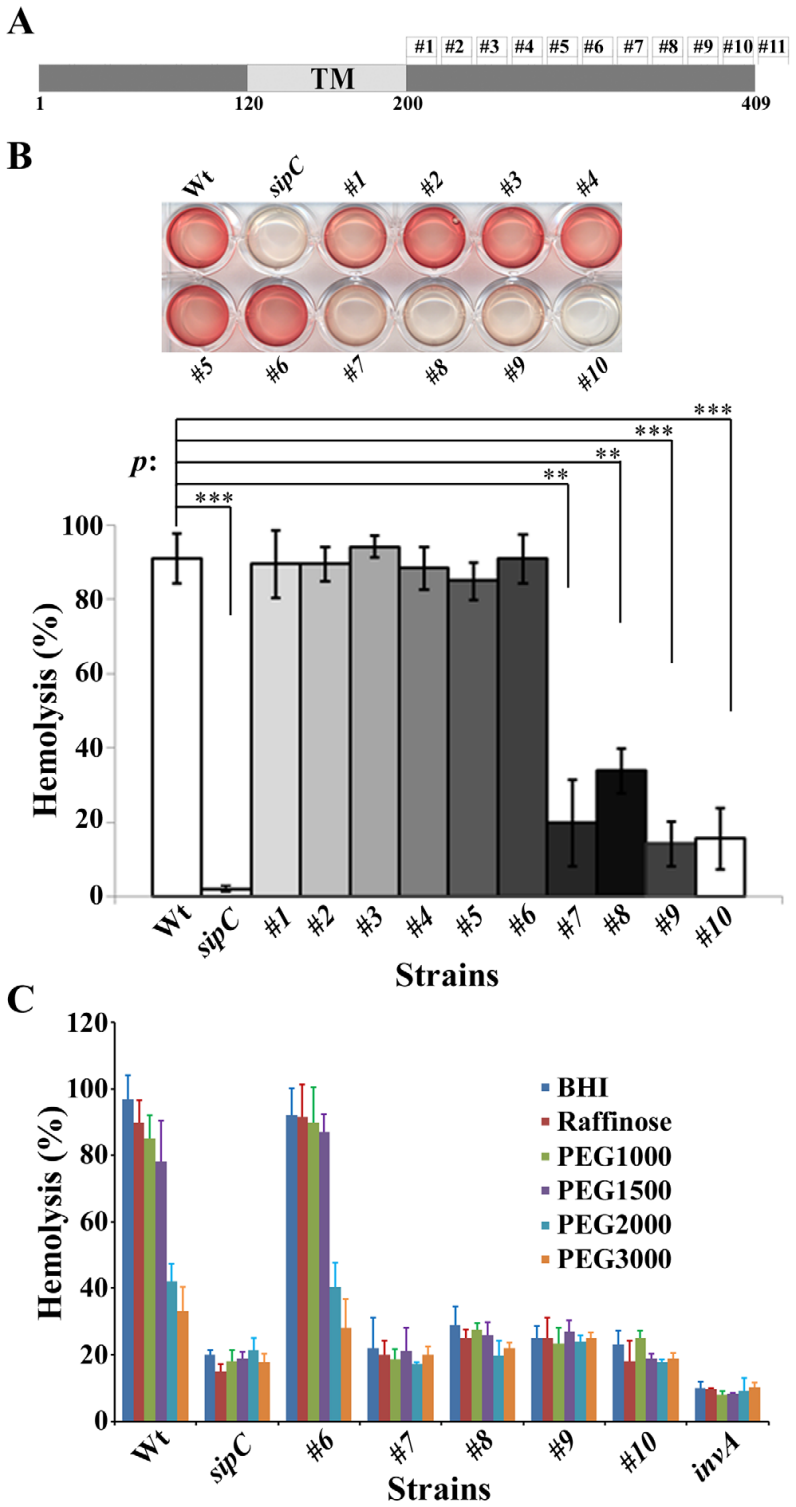
Pore forming activities of SipC C-terminal mutant strains. (**A**) Schematic diagram of the SipC protein showing the deletion/insertion sites in SipC. (**B**) Sheep Red Blood cells were infected with various *Salmonella* strains and the relative hemolysis activity was measured at OD 595 nm. The hemolysis experiments were repeated three times with the standard errors shown. Statistical analysis of hemolysis was performed by Student's t test with a *p* value of <0.05 being considered significant (*). When compared to the wild-type strain, the *p* values of *sipC*, #1, #2, #3, #4, #5, #6, #7, #8, #9, and #10 mutant strains are 0.0001(***), 0.5545, 0.5598, 0.9839, 0.2650, 0.9825, 0.9793, 0.0041(**), 0.0047(**), 0.0003(***), 0.0006(***) respectively. (**C**) The osmoprotection experiments were carried out in BHI media using the hemolysis assay described in materials and methods with BHI media alone or containing 30 mM Raffinose, PEG1000, PEG1500, PEG2000, PEG3000. The experiments were repeated three times with the standard errors shown.

SipC is approximately 43 KDa and contains a predicted 80 amino acids central hydrophobic region flanked by the N-terminal 120 amino acid residues and 209 amino acids toward the C-terminus (**Fig. 1A** and [14]). The C-terminal region has been shown to mediate the actin modulation and the effector translocation functions of SipC using the in-frame deletion/insertion replacement approach [14], [18], [19], [20]. The central hydrophobic region is predicted to play a role in membrane anchoring. The potential activities of the SipC N-terminal region may include effector secretion and/or translocation.

Type III secretion systems deliver effector proteins across the host cell membrane presumably by forming channels in the plasma membrane. This is supported by the observation that *Shigella* and *Salmonella* are hemolytic in a type III secretion-dependent manner [10], [15]. We have previously shown that the translocation activity of SipC is mediated by the C-terminus of SipC [19]. To understand whether the translocation defect observed were due to the pore forming activity, we examined the hemolytic activity of SipC C-terminal mutant #1–#10 strains [15], [19]. The SipC mutants #1 to #6 maintained wild-type levels of hemolysis while SipC mutant #7 to #10 had drastically reduced hemolysis activities (**Fig. 1B**), suggesting that this regions might be important for the pore forming activity and effector translocation.

Previous studies have demonstrated that the hemolytic lysis of RBC by *Shigella* could be prevented by addition of osmotic protectants. Osmotic protectants that are too large to pass through the bacteria-induced pore can counterbalance the intracellular osmotic pressure, leading to reduced hemolysis [10]. Molecules larger than PEG2000 (2.8 nm) can often prevent the hemolytic lysis induced by *Salmonella* [15]. To further determine whether the hemolytic activities of the *sipC* C-terminal mutant strains were due to pore formation, we performed osmoprotection experiments as previously described [15]. This assay allowed us to distinguish pore formation from other types of membrane damage that may lead to osmotic shock and hemolysis [10]. Various carbohydrates, i.e. raffinose (diameter 1.2–1.4 nm), PEG1000 (1.8 nm), PEG1500 (2.2–2.3 nm), PEG2000 (2.8 nm) and PEG3000 (3.2 nm) were added to the RBC before infection (30 mM). Consistent with the previous report [15], molecules larger than PEG1500 conferred significant protection against lysis induced by wild-type *Salmonella* as well as mutant #1–#6 strains (**Fig. 1C**). Osmoprotection was not observed when the null *sipC* strain or Mt#7–#10 strains were used, suggesting that the residual hemolysis was due to a pore larger than PEG3000 or from destabilization of the membrane by unknown mechanism similar to that reported previously [10]. Together, this data confirms the importance of the SipC C-terminus in the translocation activity, which requires the formation of pores approximately 2 nm or less.

### The N-terminal of SipC possesses domains for secretion and chaperone binding

**Figure 2:**
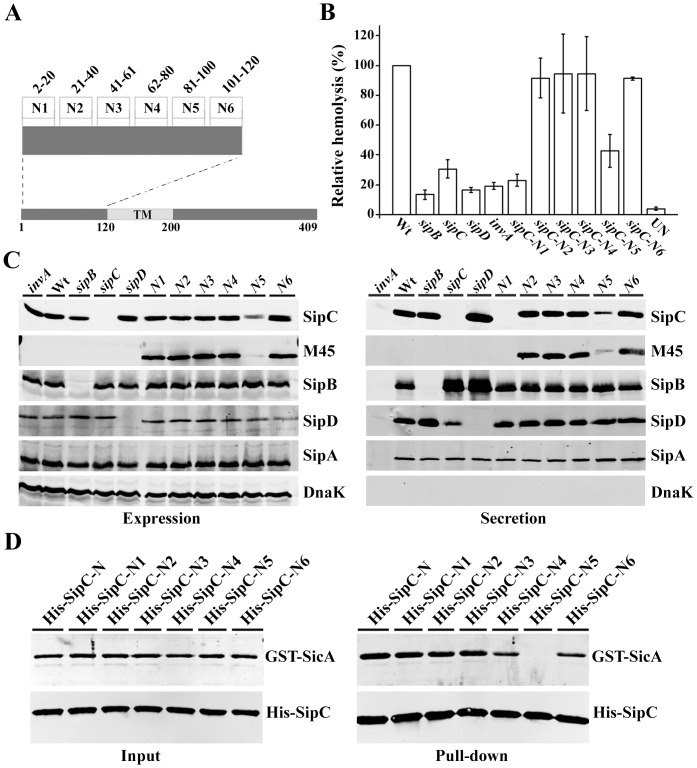
Pore forming activities of SipC N-terminal mutant strains. (**A**) Schematic diagram of the SipC N-terminus showing the deletion/insertion sites in SipC. (**B**) Sheep Red Blood cells were infected with various *Salmonella* strains and the relative hemolysis activity was measured at OD 595 nm. The hemolysis experiments were repeated three times with the standard errors shown. Statistical analysis of hemolysis was performed by Student's t test with a *p* value of <0.05 being considered significant (*). When compared to the wild-type strain, the *p* values of *sipB*, *sipC*, *sipD*, *invA*, *sipC*-N1, N2, N3, N4, N5, N6 mutant strains and the un-infected are 0.0005(***), 0.0026(**), 0.0002(***), 0.0003(***), 0.0009(***), 0.4872, 0.7540, 0.4709, 0.0124(*), 0.2500, 0.0001(***) respectively. The expression (**C**) and secretion (**D**) of SipC, SipB, SipD, SipA, and DnaK in *Salmonella* strains including the wild-type, *sipB*, *sipC*, *sipD*, *invA*, *sipC-*N1-N6. Bacterial strains were grown under SPI-1 inducing conditions and equal amounts of bacterial lysates or culture supernatants were analyzed by Western blotting using rabbit polyclonal anti-SipB, anti-SipC, anti-SipD, anti-SipA, monoclonal anti-M45 and anti-DnaK antibodies. (**D**) SipC-N5 is defective in binding to SicA. The interactions of SipC-N, SipC-N1-N6 with SicA were analyzed by pull-down assay using purified GST-SicA or GST as negative control. The presence of GST-SicA and His-SipC after pull down was determined by Western blotting with polyclonal anti-GST and monoclonal anti-His antibodies.

To fully characterize the functional region responsible for effector translocation, we made 6 in-frame deletion/insertion replacement mutations in the *Salmonella* chromosome throughout the N-terminal region of SipC (residues 1–120) (**Fig. 2A**) [19]. To understand whether the SipC N-terminal mutant strains have altered pore forming activities, we examined the hemolytic activity of SipC N-terminal mutant N1–N6 strains. The SipC mutants N2, N3, N4, and N6 maintained wild-type levels of hemolysis while SipC mutant N1 and N5 strains had drastically reduced hemolysis activities (**Fig. 2B**).

Type III effectors often harbor the secretion and translocation signals at their N-terminal regions. Thus, the reduced hemolytic activities of SipC mutant N1 and N5 strains may have altered secretion and/or translocation of type III effectors. To test whether the SipC N-terminal mutations affected the type III effector secretion, we examined the expression and secretion of SipC, SipB, SipD, and SipA proteins in these N-terminal mutant strains. Similar levels of expression and secretion were observed in *sipC-N2*, *sipC-N3*, *sipC-N4* and *sipC-N6* mutant strains (**Fig. 2C**). In contrast, the *sipC-N1* mutant strain failed to secret SipC-N1 even though its expression was comparable to that of the wild-type strain. This observation suggests that the N-terminal 20 residues are important for SipC secretion [21]. Interestingly, SipC-N5 was detected at drastically lower amounts in both the lysate and secreted fractions. Further experiments showed that the wild-type SipC-N and its derivatives were able to bind SicA, the chaperone for SipC [22], except SipC-N5 (**Fig. 2D**). Together, these data (**Fig. 1** **–** **2**) suggest that the N-terminal region (residues 1–120) possess domains for secretion and chaperone binding and most likely does not contain functional regions directly involved in effector translocation, which resides at the C-terminus of SipC.

### The C-terminal region (340–409) of SipC is required for SipB-SipC complex formation

**Figure 3:**
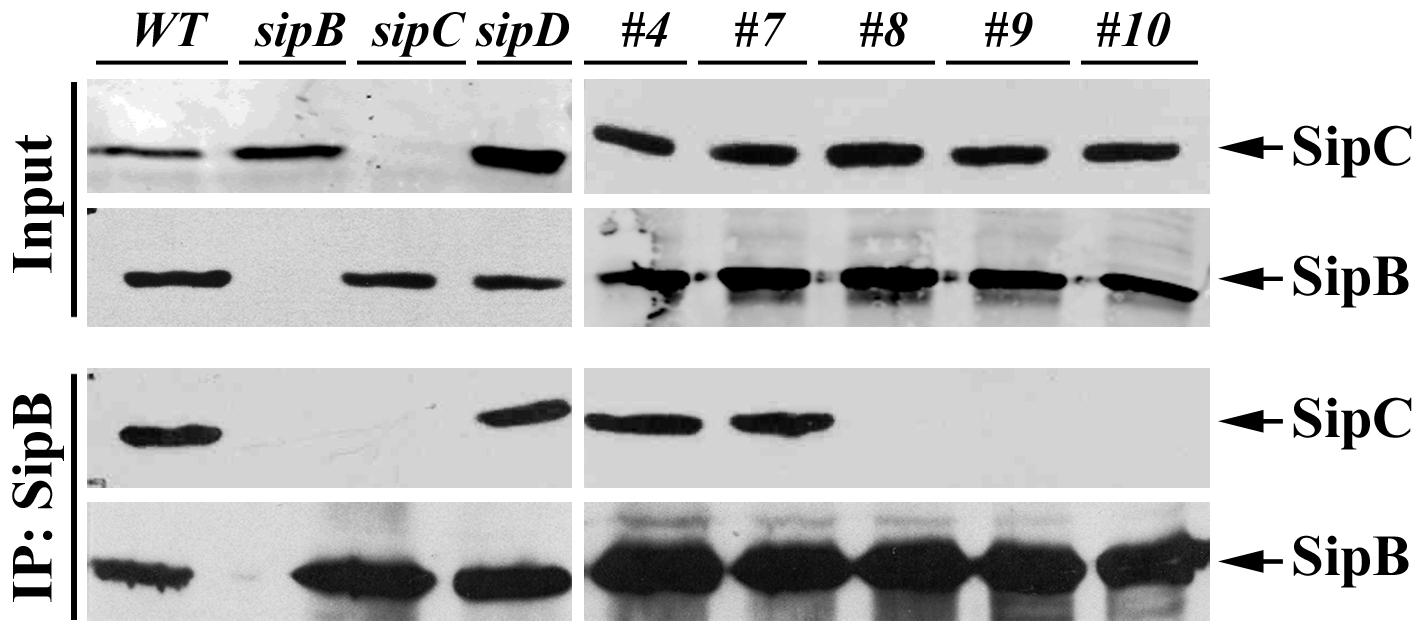
The C-terminal region (340–409) of SipC is required for SipB-SipC complex formation. Bacterial culture supernatants from various *Salmonella* strains were immunoprecipitated with antibodies to SipB and were probed by Western blotting using anti-SipB and anti-SipC antibodies.

Previous studies have reported that SipC forms an extracellular complex with SipB upon secretion [14] and recombinant SipC and SipB proteins bind to each other *in vitro* [17]. We have previously shown that the SipC C-terminal region (321–409) is required for translocation of effectors with mutant Mt#7, Mt#8, Mt#9, and Mt#10 deficient in translocation (**Fig. 1A** and [19]). We hypothesized that the loss of the translocation activities of the SipC C-terminal mutants could be partly due to their inability to bind to SipB. We therefore examined the ability of SipC C-terminal mutants to form complexes with SipB by co-immunoprecipitation using *Salmonella* culture supernatants. Consistent with a previous report [14], SipC forms a complex with SipB in culture supernatant of wild type *Salmonella* (**Fig. 3**). SipC also forms a complex with SipB in the absence of SipD (**Fig. 3**) even though SipD is required for the translocation activity. Our SipC translocation deficient mutants Mt#8, Mt#9, and Mt#10 failed to form the complex with SipB while wild type SipC and the translocation competent Mt#4 strain were still capable of binding to SipB (**Fig. 3**). Null *sipC* and *sipB* mutant strains were used as negative controls. Interestingly, SipC translocation and pore formation deficient mutant Mt#7 was still capable of forming a complex with SipB. Although it cannot be ruled out that the translocation activities of SipC and SipB are independent of their binding abilities, our data indicate that SipC and SipB interaction is important for their membrane localization and pore formation. To ensure that the SipC mutations did not significantly alter the conformation of the protein and therefore their binding activities, limited proteolysis [23] was carried out for the wild-type SipC as well as the SipC derivatives #7, #8, #9, and #10 mutants. All the SipC mutant proteins produced similar proteolytic banding patterns as that of the wild-type SipC (**Fig. S1**), suggesting that the folding of these mutated SipC proteins was not significantly impaired.

### SipC mutants that no longer interact with SipB are also defective in translocon assembly in the host cell membrane

The insertion of SipB and SipC into the host plasma membranes is believed to be a prerequisite for the formation of a channel or a pore that enables the passage of effector proteins from the bacteria into the host cell. Indirect evidence using *sipB*, *sipC*, and *sipD* null mutant strains have shown that SipB and SipC are not targeted to the host membranes in the absence of any one of these translocators [13]. The molecular events and how the SipB and SipC membrane insertion process occurs remain unknown. For example, it is not known if SipB and SipC complex formation is required for the membrane insertion during infection. The availability of SipC translocation mutants gave us the opportunity to investigate this further.

**Figure 4:**
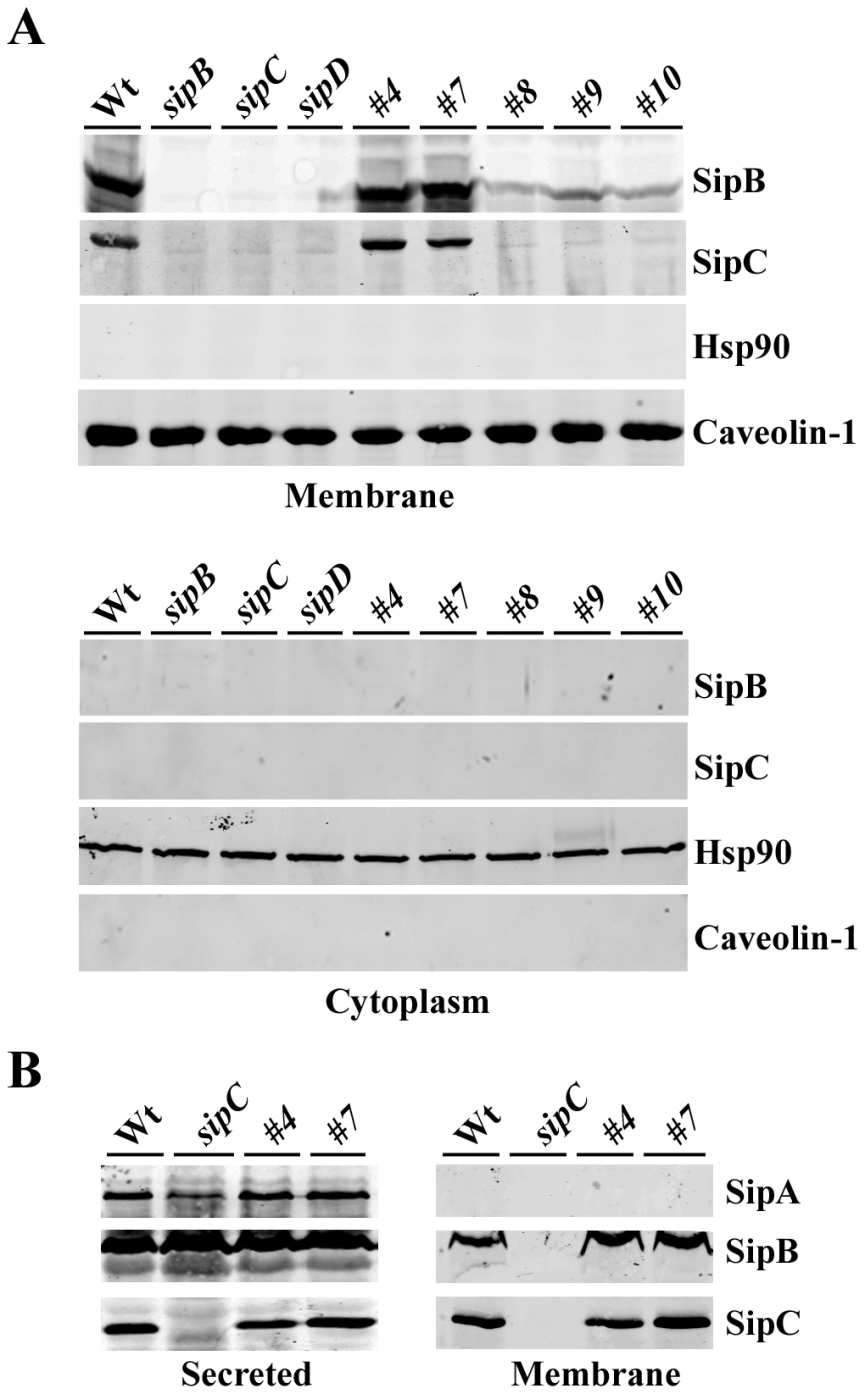
SipC mutants that no longer interact with SipB are also defective in translocon assembly in the host cell membrane. (**A**) HeLa cells were infected with the wild-type *Salmonella* strains or its mutant derivatives as indicated. Infected cells were fractionated and the membrane fraction (upper panel) or the cytosolic fraction (lower panel) were resolved by SDS-PAGE and analyzed by Western blotting with antibodies against SipB, SipC, Caveolin-1 (membrane), and Hsp90 (cytoplasmic). (**B**) HeLa cells were infected with the wild-type *Salmonella* strains or its mutant derivatives as indicated. The membrane and cytosolic fractions were similarly processed and probed with antibodies against SipA, SipB and SipC.

To evaluate the association of SipC and SipB with host cell membrane during *Salmonella* invasion, we performed cell fractionation assays using HeLa cells infected with the wild-type *Salmonella* or *sipC* mutant derivatives (**Fig. 4**). As expected, we were able to demonstrate the presence of both SipB and SipC in the membrane fractions of host cells infected with the wild-type *Salmonella*. In contrast, SipB and SipC were not detected in the membrane fractions of cells infected with the *sipB* or the *sipC* null mutant strains (**Fig. 4**). The amount of SipB and SipC in the membrane fractions of cells infected with Mt#8, Mt#9, Mt#10, or the *sipD* mutant strains were almost undetectable. Interestingly, SipB and SipC were detected in the membrane fractions of cells infected with Mt#7 strain at a level similar to that of the wild-type strain (**Fig. 4**). This was intriguing because mutant Mt#7 is defective in translocating effectors, but remains capable of forming a complex with SipB (**Fig. 3**). SipB and SipC were also detected in the membrane fractions of cells infected with Mt#4 mutant strain, which is translocation competent. Caveolin-1 was used as a control for membrane fraction while Hsp90 was used as a cytosolic marker. SipA, a type III secreted protein that is also present on the *Salmonella* surface, was used as an internal negative control to test whether *Salmonella* membrane-associated proteins could have contaminated the membrane fraction. No SipA was detected (**Fig. 4B**), indicating that there are no detectable amounts of residual *Salmonella* surface proteins in the host cell membrane fractions. This data strongly suggest that the complex formation of SipC and SipB is required for the proper host cell membrane localization of both SipC and SipB during *Salmonella* infection. The formation of functional pores requires the insertion of both SipC and SipB in the host membrane. SipC mutants #8, 9 and 10 are defective in hemolysis (**Fig. 1**), in forming complexes with SipB (**Fig. 3**), and incapable of proper insertion/localization into host membrane during infection (**Fig. 4**). However, the translocation deficient mutant #7 still binds to SipB and properly inserts into the host cell membrane. These data therefore supports the notion that the proper insertion of SipB and SipC into the host membrane requires the complex formation of SipB and SipC.

Wild type SipB and SipC proteins possess hydrophobic trans-membrane regions and are capable of associating with membranes [14], [17]. We decided to examine the abilities of the wild type SipC protein and its mutant derivatives to localize to the host cell membrane when ectopically expressed in cultured cells. Wild-type SipC protein and its mutants SipC-#4, #7, #8, #9, and #10 are all capable of associating with the host cell membranes (**Fig. S2**). This result suggests that the mutated proteins still capable of localizing to the plasma membrane when expressed in the mammalian cells.

### SipC forms a complex with SipB in *Salmonella* infected cells

**Figure 5:**
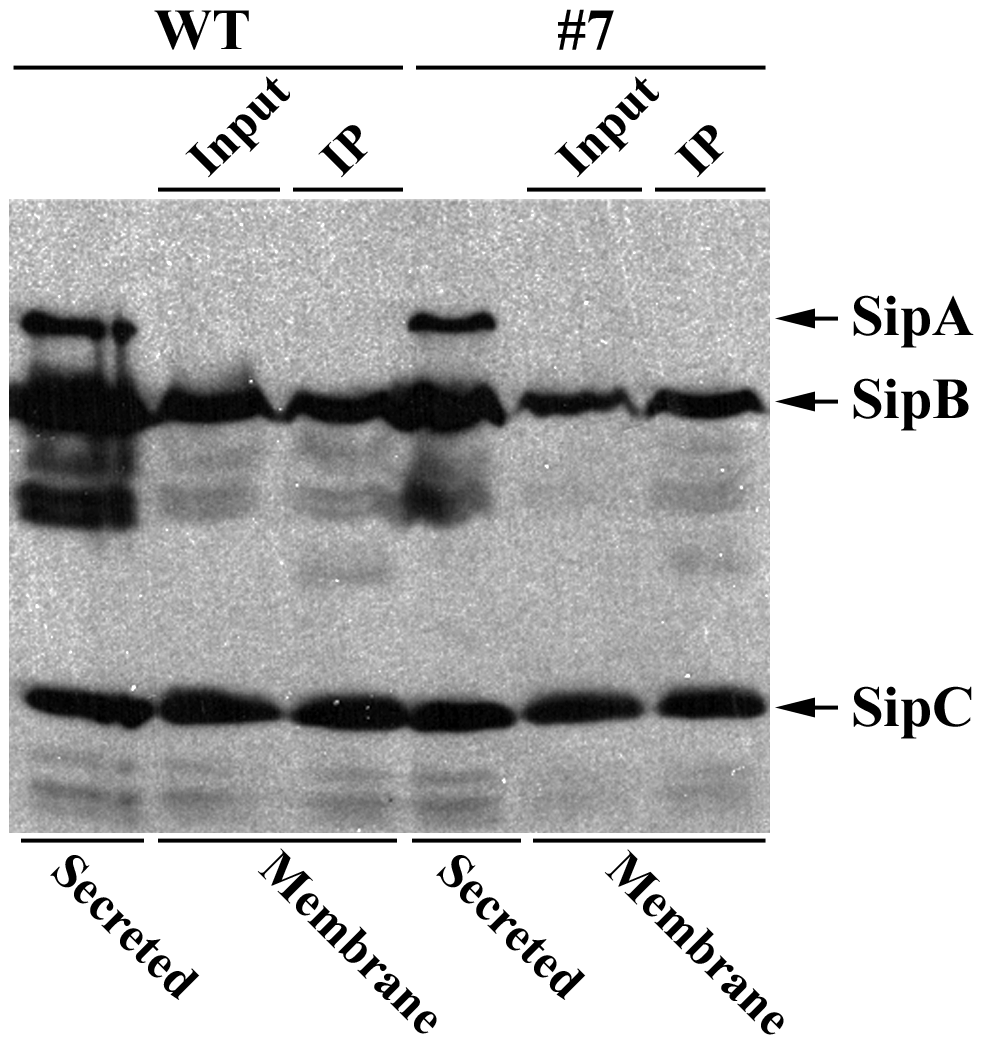
SipC forms a complex with SipB in infected cell lysates. HeLa cells were infected with wild type *Salmonella* and SipC mutant (Mt#7) strains. Infected HeLa cells were fractionated and membrane associated proteins were immunoprecipitated with SipB antibody. Immunoprecipitated samples were analyzed by SDS-PAGE and western blotting using anti-SipA, anti-SipB, and anti-SipC antibodies.

The translocation-deficient mutant #7 was impaired in pore formation and yet it remained capable of forming a complex with SipB and of associating with host cell membranes. To determine if SipB and SipC proteins translocated during infection were able to form a complex, we carried the coimmunoprecipitation assay using *Salmonella* infected cells. As shown in **Fig. 5**, SipB and SipC are found in a complex when infected with the wild-type *Salmonella* or the mutated SipC Mt#7 strain. This result illustrated that the membrane extracted SipB and SipC proteins from infected cultured cells still interact, suggesting that they may form a complex during infection for their membrane insertion/localization in host cells.

The multimerization and self-association abilities of translocator proteins have been predicted to play an important role during the translocation process [24]. To determine whether the SipC mutated proteins were still capable of self-association, clarified bacterial culture supernatants were cross-linked with the zero-length cross-linker EDC. Wild-type SipC protein and all its derivatives were still able to oligomerize as dimers, trimers, and high order oligomers in bacterial culture supernatants (data not shown). We also examined the oligomerization of SipC in HeLa cells infected with wild-type *Salmonella* or the Mt#7 mutant strain. We found that SipC Mt#7 can still form oligomers as high molecular weight species (data not shown).

## Discussion

The delivery of *Salmonella* type III effector proteins across the host cell membrane requires the formation of the translocon by SipB, SipC, and SipD. This poses great challenges since the translocator proteins are synthesized in the bacterial cytosol and have to be secreted across the bacterial membranes and subsequently be inserted into the host cell membranes. SipB and SipC contain hydrophobic regions and are inserted into the host cell membrane presumably to form a channel or pore [6], [7], [8], [9]. SipD possesses no hydrophobic regions and is not inserted into host cell membranes, but is also required for the formation of functional pores. How SipB and SipC insert into the host cell membrane is not known. We have previously reported that the SipC C-terminal region (321–409) is required for translocation of effectors [19]. We created a series of translocation-deficient mutants (Mt#7 to Mt#10) that are valuable in studying the roles of SipC in forming the translocon. We hypothesized that the loss in the translocation activities of these SipC mutants could be attributed to the loss in binding to SipB or in inserting into the host cell membrane. Thus, one key question was whether SipB and SipC complex formation is required for their insertion into the host cell membrane.

*Shigella* IpaB, the functional equivalent of *Salmonella* SipB, has been shown to associate with host membranes in the absence of IpaC [10]. This is not the case for SipB, which is not found in the host cell membranes in the absence of SipC [13]. Both SipC and SipB associate with artificial membranes *in vitro*, but do not form functional pores [17], [25]. Our SipC mutant, Mt#7, which lacks amino acids 321–340, maintained its ability to bind to SipB and was inserted into host membranes of epithelial cells. This was in contrast to SipC mutants that lack amino acids 340–409, which did not interact with SipB and were not associated with host cell membranes. This data is consistent with the notion that SipB and SipC form a complex before being inserted into the host cell membrane. We showed that the SipC mutants that lack amino acid residues 321–409 fail to form functional pores in erythrocytes using the contact hemolysis assay as an indirect indication of type III secretion system induced pore formation. Interestingly, the SipC mutant that lacks amino acids 321–340 (Mt#7) and is defective in translocon pore formation was still capable of associating with isolated RBC membranes. These findings were in line with results in tissue cultured cells that showed that SipC Mt#7 was still capable of associating host membranes. Although SipD is known to be required for forming a functional pore, we were not successful in determining any direct interactions between SipD and SipC. Therefore we cannot rule out the possibility that SipC Mt#7 has lost its inherent interacting activity with SipD, which ultimately allows SipC, SipB and SipD to form functional pores.

Previous studies have indicated that oligomeric complexes formed by the translocator proteins contribute to pore formation in *Pseudomonas* [24]. We examined the abilities of SipC and its derivatives to self-associate in bacterial culture supernatants (secreted proteins). Our data did not show any significant impairment in the oligomerization of the wild type SipC protein and its derivatives (data not shown). In addition, the wild type SipC protein and the SipC Mt#7 protein, capable of plasma-membrane association and interaction with SipB, were able to self-associate in the plasma-membrane of infected host cells (data not shown). This data suggest that the loss of translocation activity by the SipC mutants was not due to the failure of SipC to self-assemble. This data is plausible because both the C-terminal [14], [18], [19] and the N-terminal (unpublished data) truncations of SipC can self-associate. Furthermore, both domains contain the predicted coiled-coil domains that could facilitate such interactions [26], [27].

In summary, our results indicate that the SipC and SipB complex formation is essential for forming a functional translocon, possibly by inserting into host membranes as a complex during *Salmonella* infection. We propose a mechanism whereby SipC binds SipB through its C-terminal region to be in a membrane-insertion competent state to facilitate subsequent translocon formation. Our study does not completely preclude the possibility of sequential insertion of SipB and SipC into the host cell membrane. Future studies are required to gain a better understanding of how the translocon is formed during *Salmonella* infection.

## Materials and Methods

### Bacterial Strains and mammalian cell lines

Wild-type *S. typhimurium* strain SL1344 (SB300), its *sipC* (SB220), *sipB* (SB169), and *sipD* (SB221) null mutants have been previously described [6], [28]. In-frame chromosomal gene replacements in *sipC* were carried out as described before [29] by an allelic-exchange suicide vector pSB890. Chromosomal mutants N1-6 were constructed using the in-frame deletion/insertion replacement strategy similar to the Mt#7, Mt#8, Mt#9, and Mt#10 mutant construction as described previously [19]. Briefly, in-frame deletion/insertion replacement mutations in SipC (residues 1–120) with the M45-tag epitope were constructed using the polymerase chain reaction (PCR) and standard molecular biology techniques with a two-step cloning protocol [30]. Chromosomal gene replacements in SipC were carried out as described before by an allelic-exchange suicide vector pSB890 [29], [31]. The mammalian cell lines HeLa (CCL-2), HEK293T, and NIH3T3 were purchased from the ATCC cell biology stock center (Manassas, VA). HeLa and HEK293T cells were maintained in Dulbecco's modified Eagle's medium supplemented with 10% fetal bovine serum.

### Coimmunoprecipitation and Pull-down assays

*Salmonella* strains were grown under SPI-I inducing conditions as described previously [32]. Briefly, bacteria were grown overnight at 37°C in 0.3 M LB medium, diluted 1∶20 into fresh medium, and grown for another 3 hrs at a rotation speed of 200 rpm to obtain an OD_600_ of 0.8 to 0.9. Secreted proteins were recovered by three cycles of centrifugation at 50,000× g for 30 min each time and pre-cleared with TrueBlot anti-Rabbit Ig IP Beads (eBioscience). The supernatants were then incubated with rabbit polyclonal anti-SipB antibody for 2 hrs at 4°C. TrueBlot anti-Rabbit Ig IP Beads were subsequently added and the samples were incubated for an additional hour at 4°C. The beads were washed four times in lysis buffer (20 mM Tris-HCl, pH 7.5, 150 mM NaCl, 1 mM EDTA, 1% Triton X-100). The immune-complex was separated by SDS-PAGE and the proteins were detected by Western blot using the TrueBlot protocol as per manufacturer's instructions (eBioscience).

Before the pull-down assay, His- or GST-tagged recombinant proteins were pre-clarified at 100,000× g, and their concentrations were determined by the Bradford assay (Bio-Rad) using bovine serum albumin as a standard. Purified His-SipC, His-SipC-C and His-SipC-N and its derivatives were immobilized on Ni-nitrilotriacetic acid beads (NOVAGEN, Gibbstown, NJ) for 1 hr at 4°C and washed three times with cold PBS. Equal concentrations of GST or GST-SicA were added to the immobilized His fusion proteins and incubated for 2 hrs at 4°C under gentle rotation. The mixtures were subsequently washed four times in PBS containing 1% NP-40, and then eluted from the beads by addition of Laemmli loading buffer, boiled and resolved by SDS-PAGE along with pre-incubation samples (inputs) of the reactions. Samples were further analyzed by Western blotting using monoclonal anti-His antibody (Invitrogen) and rabbit polyclonal anti-GST antibody. The anti-M45 McAb used was a gift from Dr. Patrick Hearing at SUNY Stony Brook, New York.

### Chymotrypsin Digestion

Secreted SipC was prepared from bacterial cultures grown as described above. Limited proteolysis was carried out in chymotrypsin at 25°C. Samples were taken at different time intervals and digestion products were separated by SDS-PAGE and subjected to western blotting using polyclonal anti-SipC antibody.

### Cell Fractionation Assays

*Salmonella* strains were grown as described above. Cytosolic and plasma-membrane associated proteins in *Salmonella*-infected host cells were assessed as described previously [13], [33]. Briefly, HeLa cells were infected with *Salmonella* at a multiplicity of 100 for 60 min in Hanks balanced salt solution (HBSS). Infected cells were washed with PBS and incubated in Dulbecco's modified Eagle medium containing 100 µg/ml of gentamicin for 30 min at 37°C to kill extracellular bacteria. Infected cells were treated with Proteinase K (50 µgml^−1^) for 15 min at 37°C to minimize extracellular-associated secreted proteins and collected by low-speed centrifugation. To extract cytosolic proteins, cells were lysed in 50 mM Tris-HCl, pH 7.5, 1 mM PMSF, and protein inhibitor cocktail (Sigma) containing 0.2% saponin. Lysates were clarified at 20,000× g at 4°C for 60 min; supernatant fractions containing cytosolic proteins were collected for SDS-PAGE and western blot analysis. The saponin insoluble fractions containing membrane associated proteins were lysed in membrane buffer (10 mM Tris-HCl, pH 7.4, 5 mM MgCl_2_, 1 mM PMSF, and protein inhibitor cocktail (Sigma) containing 0.15 M NaCl 1% Triton X-100 for 60 min at 4°C. Membrane associated proteins were collected by centrifugation and subjected to SDS-PAGE and western blotting. The above detergent based fractionation assays to extract cytosolic and membrane associated proteins were routinely verified using the mechanical fractionation assay as previously described [34]. When indicated, transfected cells were lysed 48 hrs post-transfection as described above to extract cytosolic and then membrane associated proteins.

### Hemolysis Assay

The contact mediated hemolysis of sheep erythrocytes was performed as previously described [15]. Briefly, *Salmonella* strains were grown overnight in BHI medium at 37°C under static conditions. The bacteria were sub-cultured (1∶50) into fresh BHI medium and grown to mid-log phase. The bacteria were collected by centrifugation and resuspended in BHI. Sheep red blood cells were washed three times with PBS and resuspended in BHI (50% v/v). Equal volumes of red blood cell suspension and bacteria (1×10^9^ cfuml^−1^) were added to 24-well micro titer plates, centrifuged at 2,000× *g* for 10 min at 20°C to facilitate close contact between the bacteria and red blood cells. The red blood cells and bacterial suspensions were then incubated at 37°C for 4 hrs, resuspended in 100 µl PBS, and centrifuged at 2,000× *g* for 10 min. The supernatant fractions were transferred to a clean micro titer plate and the absorbance of released hemoglobin measured at 595 nm. For osmoprotection assays, hemolysis assays in the presence or absence of 30 mM carbohydrates raffinose, PEG1000, PEG1500, PEG2000, and PEG3000 were performed in a similar manner. The examination of SipC associated with RBC after hemolysis was performed as previously described [10] by floatation in a sucrose density gradient. Proteins associated with RBC membranes were subjected to SDS-PAGE and western blotting using anti-SipC and anti-SipB antibodies.

## Supporting Information

Figure S1 **The stability of the wild SipC protein and its derivatives assessed by limited proteolysis.** *Salmonella* culture supernatants (secreted proteins) grown under SPI-1 conditions were incubated with chymotrypsin at 25°C. Aliquots were taken from the reaction mixture at the indicated time intervals and the degradation was monitored by SDS-PAGE and Western blotting analysis with polyclonal anti-SipC antibody.
(PDF)

Figure S2 **The wild-type SipC and its mutant derivatives are targeted to the membrane when expressed in HeLa cells.** HeLa cells were transfected with plasmids expressing wild-type SipC and its mutant derivatives. 48 hrs post transfection, cells were fractionated and subjected to SDS-PAGE and Western blotting analysis with antibodies against SipC, caveoli-1 (membrane), and Hsp90 (cytoplasmic).
(PDF)
