# Supplementary figures and images for: SipB-SipC Complex Is Essential for Translocon Formation
Source: PLoS One. 2013 Mar 27;8(3):e60499. doi: 10.1371/journal.pone.0060499 (PMC3609803; doi:10.1371/journal.pone.0060499)

Fig. S1

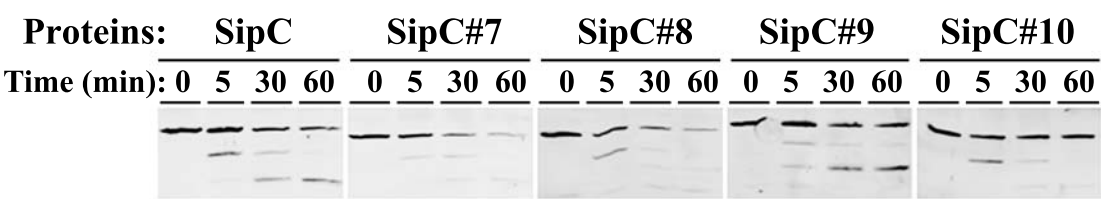

Supplement: Figure S1 — The stability of the wild SipC protein and its derivatives assessed by limited proteolysis. Salmonella culture supernatants (secreted proteins) grown under SPI-1 conditions were incubated with chymotrypsin at 25°C. Aliquots were taken from the reaction mixture at the indicated time intervals and the degradation was monitored by SDS-PAGE and Western blotting analysis with polyclonal anti-SipC antibody. (PDF) [file pone.0060499.s001.pdf]
