# Supplementary material for: SipB-SipC Complex Is Essential for Translocon Formation
Source: PLoS One. 2013 Mar 27;8(3):e60499. doi: 10.1371/journal.pone.0060499 (PMC3609803; doi:10.1371/journal.pone.0060499)
Supplement: Figure S2 — The wild-type SipC and its mutant derivatives are targeted to the membrane when expressed in HeLa cells. HeLa cells were transfected with plasmids expressing wild-type SipC and its mutant derivatives. 48 hrs post transfection, cells were fractionated and subjected to SDS-PAGE and Western blotting analysis with antibodies against SipC, caveoli-1 (membrane), and Hsp90 (cytoplasmic). (PDF) [file pone.0060499.s002.pdf]

Fig. S2

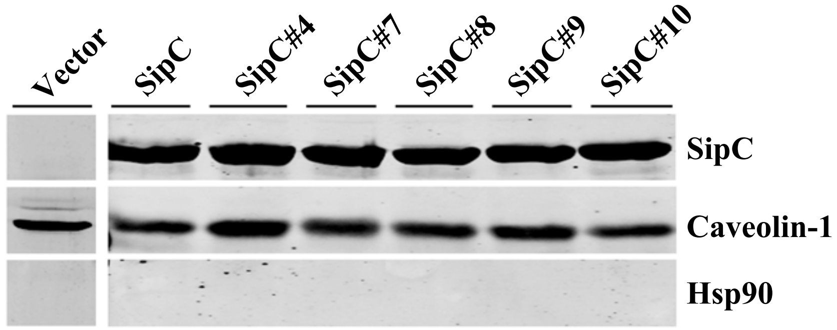
Vector
SipC
SipC#4
SipC#7
SipC#8
SipC#9
SipC#10
SipC
Caveolin-1
Hsp90
